# Characterization of single cell derived cultures of periosteal progenitor cells to ensure the cell quality for clinical application

**DOI:** 10.1371/journal.pone.0178560

**Published:** 2017-05-31

**Authors:** Stefan Stich, Alexander Loch, Su-Jin Park, Thomas Häupl, Jochen Ringe, Michael Sittinger

**Affiliations:** 1 Tissue Engineering Laboratory & Berlin-Brandenburg Center for Regenerative Therapies, Dept. of Rheumatology and Clinical Immunology, Charité - Universitätsmedizin Berlin, Berlin, Germany; 2 Department of Otorhinolaryngology, Charité - Universitätsmedizin Berlin, Berlin, Germany; 3 Dept. of Rheumatology and Clinical Immunology, Charité - Universitätsmedizin Berlin, Berlin, Germany; Augusta University, UNITED STATES

## Abstract

For clinical applications of cells and tissue engineering products it is of importance to characterize the quality of the used cells in detail. Progenitor cells from the periosteum are already routinely applied in the clinics for the regeneration of the maxillary bone. Periosteal cells have, in addition to their potential to differentiate into bone, the ability to develop into cartilage and fat. However, the question arises whether all cells isolated from periosteal biopsies are able to differentiate into all three tissue types, or whether there are subpopulations. For an efficient and approved application in bone or cartilage regeneration the clarification of this question is of interest. Therefore, 83 different clonal cultures of freshly isolated human periosteal cells derived from mastoid periosteum biopsies of 4 donors were generated and growth rates calculated. Differentiation capacities of 51 clonal cultures towards the osteogenic, the chondrogenic, and the adipogenic lineage were investigated. Histological and immunochemical stainings showed that 100% of the clonal cultures differentiated towards the osteogenic lineage, while 94.1% demonstrated chondrogenesis, and 52.9% could be stimulated to adipogenesis. For osteogenesis real-time polymerase chain reaction (PCR) of *BGLAP* and *RUNX2* and for adipogenesis of *FABP4* and *PPARG* confirmed the results. Overall, 49% of the cells exhibited a tripotent potential, 45.1% showed a bipotent potential (without adipogenic differentiation), 3.9% bipotent (without chondrogenic differentiation), and 2% possessed a unipotent osteogenic potential. In FACS analyses, no differences in the marker profile of undifferentiated clonal cultures with bi- and tripotent differentiation capacity were found. Genome-wide microarray analysis revealed 52 differentially expressed genes for clonal subpopulations with or without chondrogenic differentiation capacity, among them *DCN*, *NEDD9*, *TGFBR3*, and *TSLP*. For clinical applications of periosteal cells in bone regeneration all cells were inducible. For a chondrogenic application a fraction of 6% of the mixed population could not be induced.

## Introduction

For the regeneration of skeletal tissue defects the emerging fields of regenerative medicine and tissue engineering are becoming more and more of importance. Autologous treatments of cartilage and bone defects are currently applied methods in the clinics [1] [2]. This regeneration approach for bone and cartilage can be performed on one hand using differentiated cells like osteoblasts or chondrocytes. Autologous chondocytes are used for the treatment of joint-knee cartilage defect. A cell suspension is injected under a periosteal flap covering the defect to avoid leaking [3]. In a different approach the cells are combined with a biomaterial and placed in the defect [4]. For cartilage repair, autologous chondrocytes mixed with fibrin glue are applied to a polymer scaffold and transplanted in a cartilage defect in the knee [1]. In order to isolate these tissue specific cells, native tissue biopsies have to be taken from undamaged regions, which in turn lead to new tissue defects. To avoid the new damages, mesenchymal stem/stromal cells (MSC) or other progenitor cells are used to seed transplants and subsequently induced to form the new desired tissue [5]. One type of progenitor cells already used in clinical applications are periosteal progenitor cells. These cells are the main source for soft callus formation during fracture healing [6]. Periosteal progenitor cells are used to facilitate a cell based bone graft for the regeneration of the sinus lift [2]. Several month after transplantation an implantation of artificial teeth in the maxiliary region is possible. For this application the cells are isolated, cell culture expanded and seeded in 3D scaffolds [7]. Furthermore, periosteal progenitor cells demonstrated a promising ability to form cartilage-like tissues *in vitro*. Moreover, the ability to form lipid droplets and the gene expression of typical adipogenic marker genes was demonstrated *in vitro*, proving the multilineage capacity of the isolated cells [8, 9].

The periosteum is a thin tissue covering all bones except for the joints, which are covered by cartilage. It consists of two layers, the fibrous and the cambium layer. Latter is directly in contact with the bone and contains progenitor cells. During cell isolation from the periosteal tissue by outgrowth cultures or enzymatic digestion the chance of a mixture of cells from cambium layer and the fibrous layer is possible. The question remains if all cells isolated for these experiments and further therapeutic strategies exhibit a multilineage differentiation potential or if different cell subtypes co-exist, which are only able to differentiate into one, two or even no lineage at all. Clonal analysis of periosteal cells cultivated from seven clonal cultures derived from four donors could only display the presence of the multipotent cells in these cultures so far [8]. But the number of clonal cultures was limited in this study.

Therefore, in our new approach we wanted to characterize the cells isolated from the periosteum more detailed in clonal cultures. Besides the recording of growth kinetics, the multilineage differentiation capacity, genome-wide microarray analyses to find differently expressed genes in distinct subpopulations of clonal cultures, and fluorescence-activated cell sorting (FACS) analyses of selected surface marker were performed.

## Materials and methods

### Ethics statement

All subjects participating in this study provided written informed consent to participate in this study, which was approved by the local ethical committee of the Charité—Universitaetsmedizin Berlin.

### Isolation and cell expansion of human periosteal cells

Periosteal cells were isolated from mastoid autografts (0.5 cm^2^) taken from four independent donors undergoing mastoidectomy according to a method previously described [7]. In brief, the periosteal flap was rinsed with Hanks solution (Biochrom, Berlin, Germany) three times, minced and digested for 3 hours in Dulbecco’s modified eagle medium (DMEM)/Ham’s F12 medium (Biochrom) containing 10,000 U/ml collagenase II (Biochrom), 10% human allogenic serum (German Red Cross, Berlin, Germany), 2.5% Hepes (Biochrom) and 1% penicillin/streptomycin solution (Biochrom). Subsequently, the cells were harvested, resuspended in DMEM/Ham’sF12 medium containing 10% human allogenic serum, plated in cell culture dishes (∅ = 15 cm), and allowed to attach for about 4–6 days.

### Generation of clonal cultures

Single cells with space of 2 microscopic view fields were selected for clonal culturing. To separate single cells, clonal cylinders (Sigma-Aldrich, Munich, Germany) were placed. The cylinders were coated with silicon grease (Corning, Wiesbaden, Germany) on the lower site to eliminate medium exchange between clonal cultures and the rest of the plate. The success of isolating a single cell was confirmed microscopically. Non-adherent cells were removed by exchange of medium. Clonal growing periosteal cells were cultured under standard cell culture conditions and the medium was replaced every 2–3 days in the cylinders. When reaching about 90% confluence, clonal cultures were sub-cultured by trypsin-EDTA (Biochrom) treatment (0.5%) and subsequently replated in a well of a 6-well plate and further cultivated.

### Cell differentiation of clonal cultures

In order to demonstrate the differentiation potential of human clonal periosteal cell cultures (Passage 4) modified standard protocols that promote MSC differentiation were applied [10]. For the osteogenic induction, 5000 periosteal cells/cm^2^ were seeded and induced in DMEM/Ham’s F12 (5% human serum, 2.5% Hepes, 1% penicillin/streptomycin) containing 100 nM dexamethasone (Sigma), 0.05 mM L-ascorbic acid 2-phosphate (Sigma), and 10 mM β-glycerophosphate (Sigma). Controls were treated with DMEM/Ham’s F12 (5% human serum, 2.5% Hepes, 1% penicillin/streptomycin). For the chondrogenic induction pelleted PC micromass cultures consisting of 2.5 x 10^5^ cells were cultivated under serum-free conditions in a defined medium containing DMEM (4.5 g/l glucose) (Biochrom), ITS+1 (Sigma), 1 mM sodium pyruvate (Sigma), 100 nM dexamethasone, 0.17 mM L-ascorbic acid 2-phosphate and 10 ng/ml transforming growth factor-β3 (TGF-β3) (R&D Systems, Wiesbaden, Germany). Controls were treated with the same medium without TGF-β3. For the adipogenic differentiation, 5000 PC/cm^2^ were seeded. After 5 days after reaching confluence, cells were treated with DMEM (4.5 g/l glucose) supplemented with 10% human serum, 1 μM dexamethasone, 0.2 mM indomethacin (Sigma), 10 μg/ml insulin (Novo Nordisk, Mainz, Germany), 0.5 mM 3-isobutyl-1-methylxanthine (Sigma), and maintenance medium containing DMEM, human serum and 10 μg/ml insulin, within three cycles (3 day induction, 2 day maintenance). The controls were treated with maintenance medium only.

### Histological methods and immunochemistry

Cryosections (6 μm thick) were obtained from native tissue samples and stained with Hematoxylin (Merck, Darmstadt, Germany) for 10 min. For the demonstration of the osteogenic differentiation cells were fixed with methanol for 30 min at -20°C. The expression of alkaline phosphatase was visualized by staining with SigmaFast BCIP/NBT (Sigma) for 10 min. Von Kossa staining was used to assess the deposition of a bone specific mineralized matrix (5% silver nitrate solution (Sigma) for 30 min and after washing 5% sodium carbonate solution (Sigma) for 5 min both in darkness). To prove chondrogenic induction, increase of cartilage proteoglycans was histologically shown on 6 μm cryosections by alcian blue 8GS (Sigma) staining at pH 2.5 for 30 min. Cells were counterstained using nuclear fast red (Sigma) for 5 min. To demonstrate the increase of collagen type II production immunochemical staining was performed using the EnVision^™^+System, Peroxidase (AEC) Mouse Kit (Dako, Hamburg, Germany). Cryosections were incubated for 1 h with primary rabbit anti-human type II collagen antibodies (DPC-Biermann, Bad Nauheim, Germany) at 37°C. Subsequently, samples were treated according to the manufacturer’s protocol and counterstained with hematoxylin (Merck). Adipogenic differentiation was visualized by using a vital staining of neutral triglycerides and lipids with oil red O (Sigma) for 30min.

### Polymerase chain reaction

To demonstrate osteogenesis and adipogenesis on the messenger ribonucleic acid (mRNA) level cell lysis and total cellular RNA isolation was performed using RNeasy Mini Kit (Qiagen, Hilden, Germany) according to the manufacture's protocol. Subsequently, 2 μg total RNA was reverse transcribed with the iScript cDNA Synthesis Kit (BioRad, München, Germany) according to the manufacturer’s instructions. The housekeeping gene *Glyceraldehyd-3-Phosphate Dehydrogenase (GAPDH)* was used to normalize marker gene expression in each run. Real-time polymerase chain reaction (PCR) using the iCycler system (BioRad) was performed with titrated amounts of the cDNA samples and TaqMan Oligonucleotides, Probes and TaqMan Master Mix (Applied Biosystems, Darmstadt, Germany). For all genes listed in Table 1 following PCR conditions were performed: hot start enzyme activation at 95°C for 10 min, 40 cycles of denaturation at 95°C for 15 s, and annealing of oligonucleotides for 60 s at 60°C. Relative quantitation of marker genes was performed as described [9] and is given as percentage of the *GAPDH* product. Statistical significance was calculated with SigmaStat Software 3.5 (Systat Software GmbH, Erkrath, Germany) by using the t-test for statistical significance of gene expression.

**Table 1:** Taqman probes for real-time RT-PCR analysis.

Gene	Company	Cat. No.
*BGLAP*	Applied Biosystems	Hs00609452_g1
*FABP4*	Applied Biosystems	Hs01086177_m1
*GAPDH*	Applied Biosystems	Hs99999905_m1
*PPARG*	Applied Biosystems	Hs01115513_m1
*RUNX2*	Applied Biosystems	Hs00298328_s1

### FACS analysis

Single cell suspensions of clonal periosteal cell cultures with different differentiation potential were washed in PBS/0.5%BSA [11]. Cells were incubated with titrated primary staining reagents for 15 min on ice (2.5 x 10^5^ cells/0.1ml in PBS/0.5%BSA). Fluorescein isothiocyanate (FITC) labelled mouse anti-human CD105 (endoglin; SH-2) was purchased by Acris Antibodies (Acris Antibodies, Hiddenhausen, Germany). FITC labeled mouse anti-human CD44, CD45, and CD90 (Thy-1), and R-Phycoerythrin (PE) labeled mouse anti-human CD14, CD34, CD73 (SH-3), and CD166 (ALCAM) were purchased from Pharmingen (Heidelberg, Germany). Prior to the analysis in a FACS-Calibur cytometer (Becton Dickinson, Heidelberg, Germany), dead cells and debris were stained with propidium iodide PI (Sigma) and excluded. Data were evaluated using CellQuest Pro 6.0 software (Becton Dickinson).

### Genome-wide gene expression profiling

In order to analyse the expression of differentially regulated genes in clonal cultures with different gene expression genome-wide microarray analysis with the Affymetrix HG-U133 plus 2.0 array (Affymetrix, Santa Clara, USA) were performed for undifferentiated cells of 21 clonal cultures derived of single cells of 3 donors at the end of passage 3. Cell lysis and total cellular RNA isolation was performed using RNeasy Mini Kit (Qiagen) according to the manufacture's protocol. Gene expression analysis was performed according to the manufacturer’s recommendations. To synthesize biotin-labeled cRNA 2 μg of total RNA was used. Following fragmentation, 10 μg cRNA were hybridized on gene chips for 16h at 45°C. After washing and staining, the gene chips were scanned with the GeneArray scanner controlled by Affymetrix GCOS 1.4 software. Finally, using the Affymetrix GCOS 1.4 software and the multiarray analysis (RMA) [12] raw gene expression data were processed and normalized. Data of clonal cultures with different differentiation potential was compared and genes with a significant change call in gene expression in more than 75% of all comparisons according to GCOS software and a mean fold change (FC_m_) of >2 or <-2 were selected.

## Results

### Isolation of human periosteal cells, generation of clonal cultures and cell expansion

**Fig 1:**
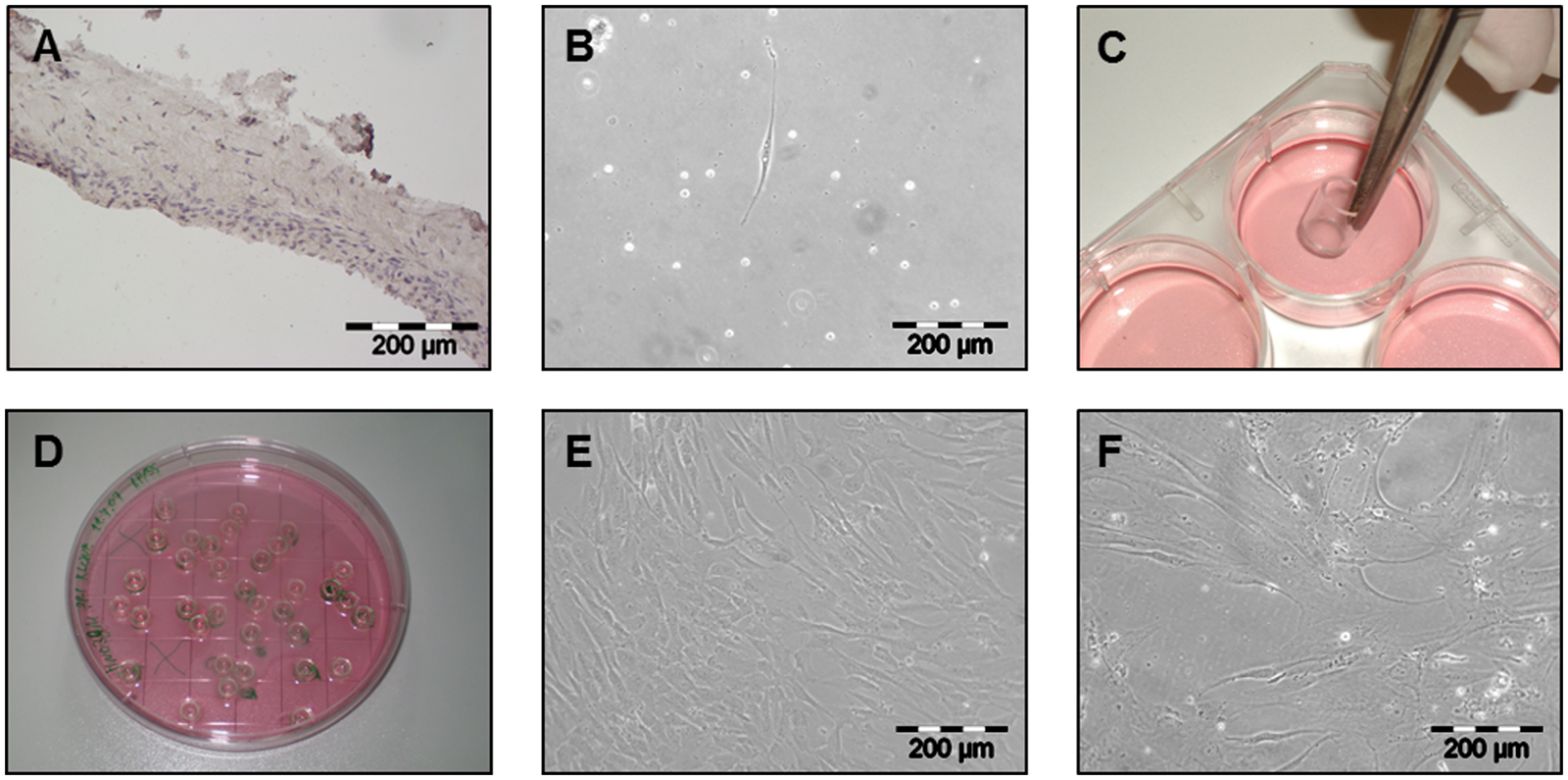
Isolation of single cells and cultivation of clonal cultures. Hematoxylin staining of native periosteal tissue (A). Single periosteal cell in cell culture 4 days after enzymatic digestion of the native tissue (B) followed by a separation using cloning cylinders (C,D). Confluent monolayer culture of clonal periosteal cells in passage 1 at day 5 (E) and in passage 9 at day 7 (F); A, B, E, F: 100x magnification.

For the generation of clonal cultures of periosteum derived cells, mastoid periosteum biopsies of 4 donors (age: mean = 39.5 years; donor 1: female, 27 years; donor 2: male, 43 years; donor 3: female, 34 years; donor 4: male, 54 years) undergoing mastoid ectomy were obtained. Hematoxylin/Eosin staining of native periosteal tissue revealed the difference of the thin, cell-rich cambium layer and the fibrous layer (Fig 1A). After isolation, cells were allowed to adhere for 5 days. Single cells with at least space of two microscopic view-fields in every direction were selected for clonal analysis (Fig 1B). Clonal cylinders were placed to separate the single cells from the remaining cells (Fig 1C and 1D). Cells were allowed to proliferate until 90% confluence and subsequently transferred to larger cell culture dishes (Fig 1E). They showed a long, stretched morphology (Fig 1B and 1E). After the second harvesting the cells were counted for the first time. Cell expansion and counting was continued until no doubling of cells was registered between seeding and harvesting. After the 3^rd^ passage only a small amount of cells was further expanded in a small cell culture flasks with a surface area of 25 cm^2^. A theoretical cell number was calculated by extrapolation to the maximal possible seeding cell number and the equivalent extrapolated harvesting cell number. In late passages cells demonstrated a flat, spread and lumpy morphology and resulted in lower cell numbers (Fig 1F). From all 4 donors 72,2% (83 out of 115) of the selected single cells were able to be expanded (53.3% - 8 of 15 from Donor 1, 80.8% - 21 of 26 from Donor 2, 64.1% - 25 of 39 from Donor 3, and 82.9% - 29 of 35 from Donor 4). The maximum proliferation of the cells varied between Passage 3 and 13 (Table 2) with corresponding cell numbers between 1.50 x 10^5^ and 7.34 x 10^10^ (17,2–36,1 population doublings) and average growth rates between 0.018 and 0.349 /day (respectively a population doubling time of 38.8 days and 2days). In order to compare the clonal cultures according to their growth features (average growth rate and maximal passage number) and the differentiation, the clonal cultures were devided in nine classes (fast μ>0.22/day, mean μ = 0.15–0.22/day and slow growth rate μ<0.15/day in any combination with the maximal passage number, high P_max_>8, mean P_max_ = 5–8 and low P_max_<5) (Tables 3 and 4). The 0.15/day corresponds to a population doubling time of 4.62 days and 0.22/day of 3.15 days. Most of the clonal cultures showed a mean passage number (class 4–6) with donor 3 cells mostly located in class 4 (fast growing). Clonal cultures with low passage numbers (class 7–9) were not found in cultures of donor 2. The majority of clonal cultures only reaching a low passage number also showed a slow growth rate and a high population doubling time. Clonal cultures with high passage number were found in all three donors but none of them in class 3 (μ<0.15/days).

**Table 2:** Overview of clonal culture growth.

	Number of clonal cylinders	Number of grown clonal cultures	% grown clonal cultures	Minimal passage of clonal cultures of a donor P_min_	Maximal passage of clonal cultures of a donor P_max_
**Donor 1**	15	8	53,3	3	8
**Donor 2**	26	21	80,8	5	10
**Donor 3**	39	25	64,1	4	9
**Donor 4**	35	29	82,9	3	13
**Overall**	115	83	72,2	3	13

**Table 3:** Classification of clonal cultures according to the maximal passage number P_max_ and the mean growth rate μ.

	high passage number P_max_>8	mean passage number P_max_ = 5–8	low passage number P_max_<5
**fast growth rate** **μ>0.22/d**	class 1	class 4	class 7
**mean growth rate** **μ = 0.15–0.22/d**	class 2	class 5	class 8
**slow growth rate** **μ<0.15/d**	class 3	class 6	class 9

**Table 4:** Distribution of clonal cultures of the 4 donors according to their growth classes.

Class	1	2	3	4	5	6	7	8	9
**Donor 1**	0	2	0	0	4	1	0	1	0
**Donor 2**	2	2	0	1	9	7	0	0	0
**Donor 3**	2	1	0	12	1	3	2	0	4
**Donor 4**	1	1	0	5	6	9	0	1	6
**Sum**	5	6	0	18	20	20	2	2	10

Detailed information of each clonal culture is given in the supporting part (S1 Table).

### Cell differentiation of clonal cultures

**Fig 2:**
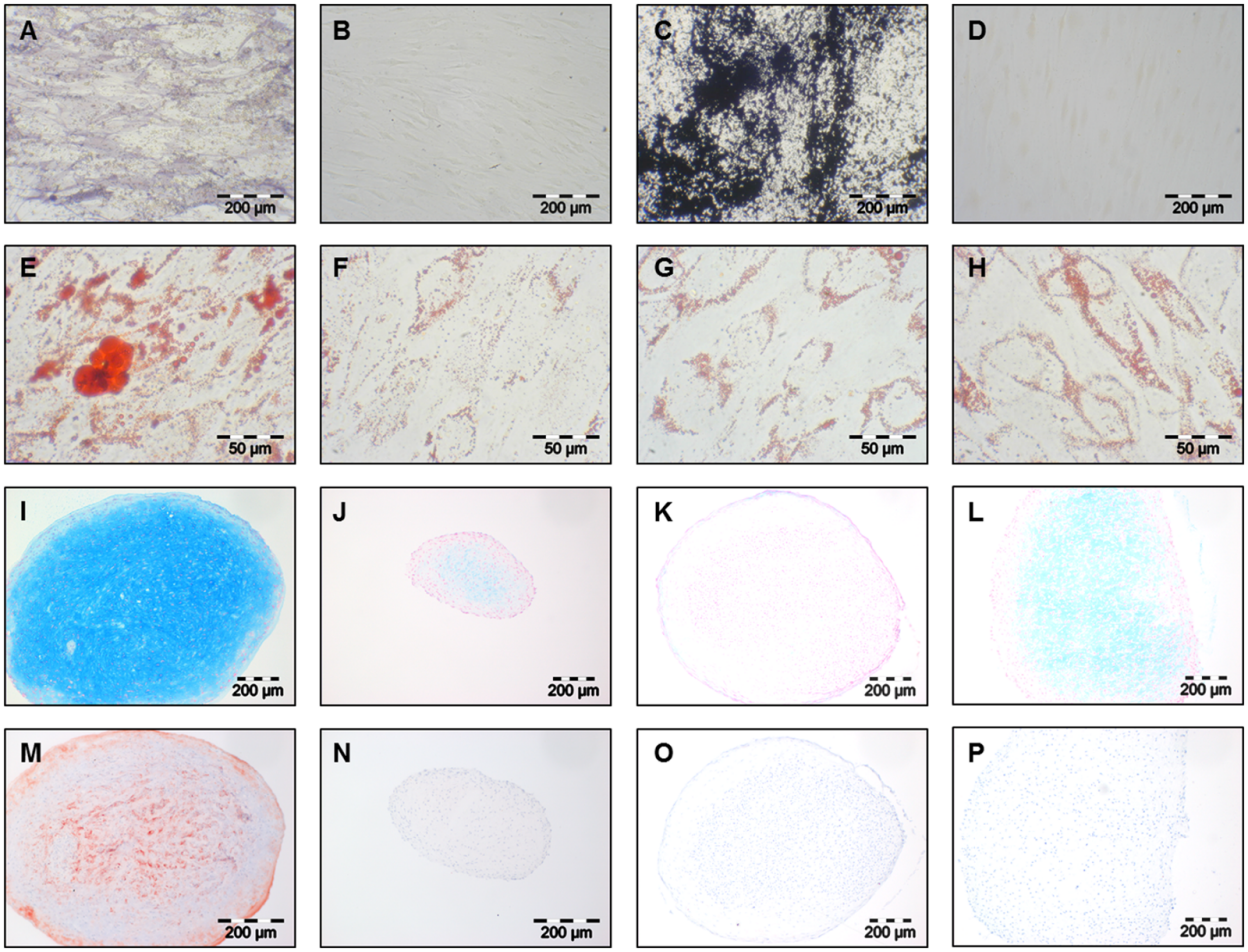
Histological and immunochemical stainings of osteo-, adipo- and chondrogenically induced clonal cultures. Alkaline phospahtase staining of osteogenically induced clonal cultures (A) and uninduced contols (B); Von Kossa staining of osteogenically induced clonal cultures (C) and uninduced contols (D); Oil red O staining of adipogenically inducible (E) and non-inducible (G) clonal cultures and corresponding uninduced controls (F,H); Alcian blue staining of chondrogenically inducible (I) and non-inducible (K) clonal cultures and corresponding uninduced controls (J,L); Collagen Type II immunochemical staining of chondrogenically inducible (M) and non-inducible (O) clonal cultures and corresponding uninduced controls (N,P); A-D and I-P 100x magnification, E-H 400x magnification.

**Fig 3:**
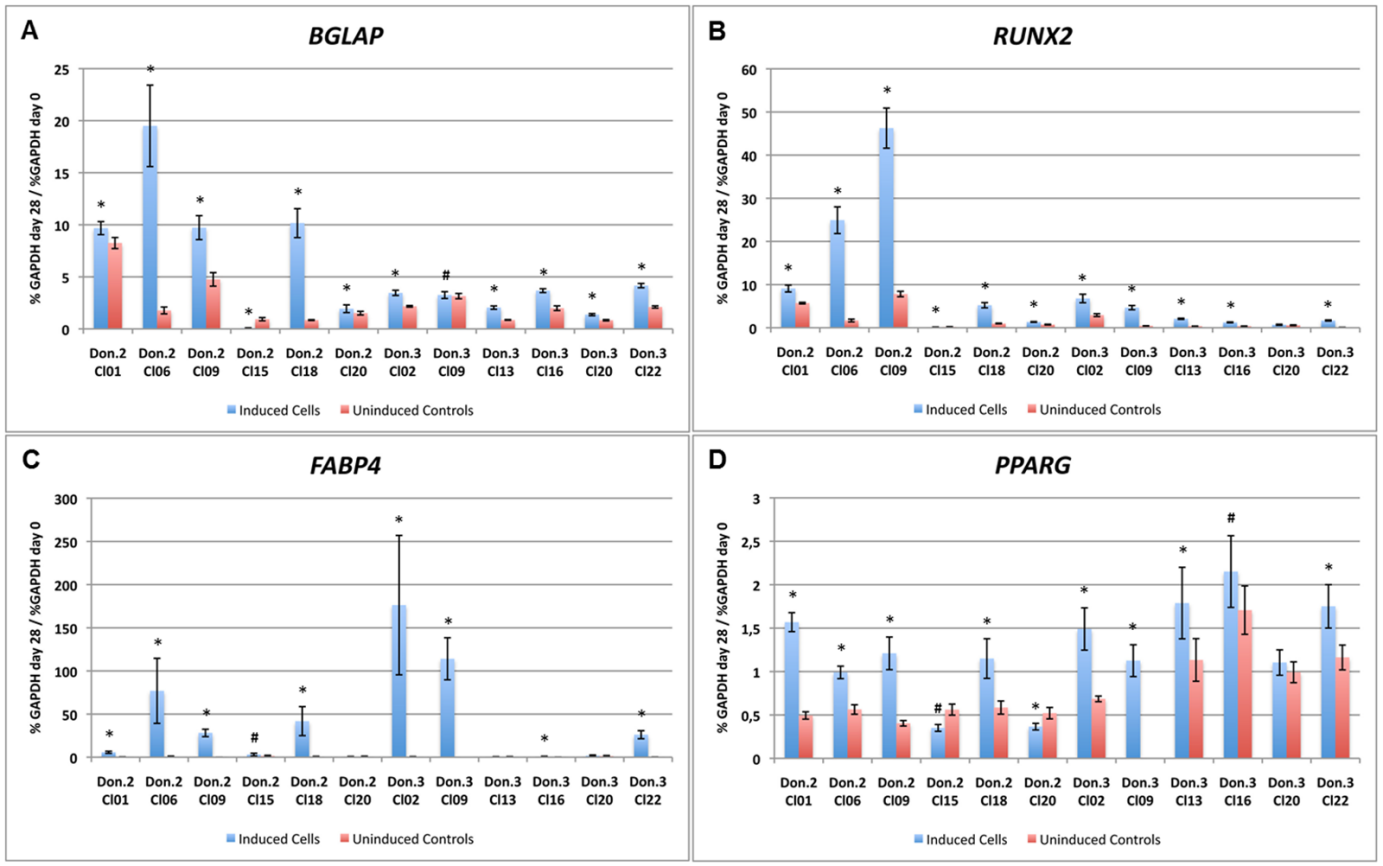
Real-time PCR of osteogenically and adipogenically differentiated clonal cultures. Osteogenic induction of clonal cultures was confirmed by gene expression of *BGLAP* and *RUNX2*. Adipogenic induction of clonal cultures was demonstrated by *FABP4* and *PPARG* gene expression. Target gene expression is given as a percentage of *GAPDH* gene expression; significant difference of induced and uninduced samples: p*≤0.001, p^#^≤0.05.

All clonal cultures that delivered a sufficient cell number for multilineage differentiation after passage 4 were induced to the osteogenic, adipogenic and chondrogenic lineage. Overall, 51 out of the 83 clonal cultures of the 4 different donors were differentiated (donor 1–3 clonal cultures—Cl2, Cl6, and Cl7, donor 2–19 clonal cultures—Cl1, Cl2, Cl3, Cl4, Cl5, Cl6, Cl7, Cl8, Cl9, Cl10, Cl11, Cl13, Cl15, Cl16, Cl17, Cl18, Cl19, Cl20, and Cl21, donor 3–19 clonal cultures—Cl1, Cl2, Cl3, Cl5, Cl7, Cl8, Cl9, Cl10, Cl11, Cl12, Cl13, Cl15, Cl16, Cl19, Cl20, Cl21, Cl22, Cl23, and Cl25, and donor 4–10 clonal cultures—Cl1, Cl4, Cl5, Cl9, Cl11, Cl12, Cl13, Cl15,Cl16, and Cl18). Alkaline phosphatase staining showed in all osteogenically induced clonal cultures an increased activity (Fig 2A) whereas non-induced controls showed no or a weak enzymatic activity (Fig 2B) after 28 days of induction. Furthermore, von Kossa staining revealed the production of a calcified extracellular matrix in all osteogenically induced clonal cultures after 28 days (Fig 2C). The non-induced controls showed no signs of matrix production (Fig 2D). In order to verify the staining results real-time PCR was performed. Due to the limited number of cells in clonal cultures it was only conducted for 12 clonal cultures (6 of Donor 2, 6 of Donor 3) for *Bone Gamma-Carboxyglutamate Protein* (*BGLAP*) (Fig 3A) and *Runt Related Tanscription Factor 2* (*RUNX2*) (Fig 3B) expression. All clonal cultures showed a significantly induced gene expression (*p≤0,001, ^#^p≤0,05) in induced cultures compared to uninduced controls at day 28. Only clonal culture 20 of donor 3 showed no significantly different *RUNX2* expression.

A successful adipogenic differentiation was found in 27 induced clonal cultures. Oil Red O staining revealed an increased accumulation of large lipid droplets (Fig 2E) while non-induced controls showed only a slight background staining after 15 days (Fig 2F). In 24 clonal cultures no difference between induced and non-induced samples was observed. Only the background staining was visible and comparable in both groups (Fig 2G and 2H). In order to verify the staining results real-time PCR was performed for the same 12 clonal cultures already tested for osteogenic differentiation for the gene expression of *Fatty Acid Binding Protein 4* (*FABP4*) and *Peroxisome Proliferator Activated Receptor Gamma* (*PPARG*) at day 15. Clonal cultures 1, 6, 9, and 18 of donor 2 and clonal cultures 15, 20, and 22 of donor 3 already demonstrated a successful adipogenic differentiation on histological level, whereas clonal cultures 15 and 20 of donor 2 and clonal cultures 13, 16, and 20 of donor 3 remained similar to undifferentiated controls. All previously Oil Red O stained samples also demonstrated a significantly higher gene expression (*p≤0,001, ^#^p≤0,05) in induced samples compared to uninduced controls for both *FABP4* (Fig 3C) and *PPARG* (Fig 3D). Clonal culture 15 of donor 2 and clonal culture 16 of donor 3 showed a very low, but significant gene expression for *FABP4* (Fig 3C). For *PPARG* expression of clonal cultures 15 and 20 of donor 2 the uninduced controls demonstrated a significantly higher expression than the induced controls (Fig 3D). Clonal cultures 13 and 16 of donor 3 showed a slightly higher gene expression of *PPARG* in induced samples whereas clonal culture 20 revealed no difference between induced and uninduced samples (Fig 3D).

To proof chondrogenesis in the periosteal cell pellet system alcian blue staining for the detection of acidic glycosaminoglycans and immunochemical staining of produced collagen type II was performed. Out of the 51 clonal cultures 49 showed acidic glycosaminoglycan production after 28 days in induced cultures (Fig 2I). Even the non-induced cultures presented a weak staining but not in an extent of the induced once (Fig 2J). Two cultures demonstrated no staining in the induced samples (Fig 2K). Here only the non-induced samples showed a weak staining similar to other non-induced clonal cultures (Fig 2L). The immunochemical collagen type II staining showed weak to strong red colour level as a proof of collagen type II production in 48 induced clonal cultures (Fig 2M). Most of the non-induced pellets showed no signs of differentiation (Fig 2N). In 6 clonal cultures the non-induced samples showed a weak collagen type II production but not to the same extent as the corresponding induced samples. Three of the 51 clonal cultures showed no collagen type II production in both induced and non-induced samples (Fig 2O and 2P). Two of them already failed in the production of acidic glycosaminoglycans. A chondrogenic induction was counted as successful only if both stainings showed a positive result.

In summary, we could demonstrate that all 51 clonal cultures showed an osteogenic and 48 exibited a chondrogenic differentiation capacity, whereas only 27 revealed an adipogenic *in vitro* differentiation capacity. This led to 25 clonal cultures with a differentiation competence into all the lineages, 23 with an osteo- and chondrogenic, 2 with an osteo- and adipogenic, and 1 only an osteogenic differentiation capacity (Table 5). When comparing the differentiation capacity of the clonal cultures from each donor quantitatively, it is shown that all donors contained clonal cultures differentiating towards the osteo-, chondro-, and adipogenic lineage and also clonal cultures differentiating towards the osteo- and chondro lineage. Donor 4 developed the most clonal cultures with a tripotent differentiation potential (8) compared the bipotent clonal cultures (2). The ratio of clonal cultures from donor 2 is also in advantage of tripotent cells (10/8) but demonstrated one clonal culture differentiating towards the osteo- and adipogenic lineage. Clonal cultures from donor 1 showed one tripotent clonal culture and two with an osteo-, chondogenic differentiation capacity. Clonal culture of donor the revealed the most mixed population. Here, only 6 clonal cultures had a tripotent differentiation potential but 10 an osteo-, chondogenic differentiation capacity. Also one clonal culture was differentiating towards the osteo- and adipogenic lineage. Additionally, from this donor the only clonal culture solely differentiating toward lineage was found. Detailed information for each clonal culture is given in the supporting part (S2 Table).

**Table 5:** Overview of the differentiation potential of all investigated 51 clonal cultures.

		O/C/A	O/C	O/A	C/A	O	C	A
**Donor 1**	3	1	2	-	-	-	-	-
**Donor 2**	19	10	8	1	-	-	-	-
**Donor 3**	19	6	11	1	-	1	-	-
**Donor 4**	10	8	2	-	-	-	-	-
**Sum**	51	25	23	2	-	1	-	-
**Rate in %**		49.0	45.1	3.9	-	2.0	-	-

Multipotent: O/C/A, bipotent: O/C, O/A or C/A, unipotent: O, C or A. O: Osteogenesis, C: Chondrogenesis, A: Adipogenesis.

Comparing the growth characteristics of the tested clonal cultures (Tables 3 and 4) with differentiation capacity (S2 Table), it is clear that all clonal cultures of class 1 and 2 (P_max_>8, μ>0,22/d or μ = 0,15–0,22/d) could be differentiated. For clonal cultures with a mean P_max_ of 5–8 not all clonal cultures of all three classes could be differentiated (class 4 = 17 of 18, class 5 = 15 of 20, class 6 = 7 of 20). One of the two clonal cultures of class 7 could also be differentiated. None clonal culture of class 8 and 9 reached passage 4 or delivered enough cells to initiate the differentiation. Regarding the differentiated clonal cultures, 27 were found to able to differentiate towards the adipogenic lineage and 24 were not able. Comparing their growth characteristics, 2 of 5 class 1 clonal cultures, 3 of 6 class 2 clonal cultures, 10 of 17 class 4 clonal cultures, 9 of 15 class 5 clonal cultures, and 3 of 7 class 6 clonal cultures were able to differentiated adipogenically. The other cultures from the classes and the one clonal culture of class 7 were not able to do so. There seems to be no correlation of growth behaviour and differentiation capacity. When beholding the 3 clonal cultures that did not differentiate toward the chondrogenic lineage it also appears that there is not correlation to the growth characteristics. These 3 belong to the very different classes 1, 4, and 6.

### FACS analysis

**Fig 4:**
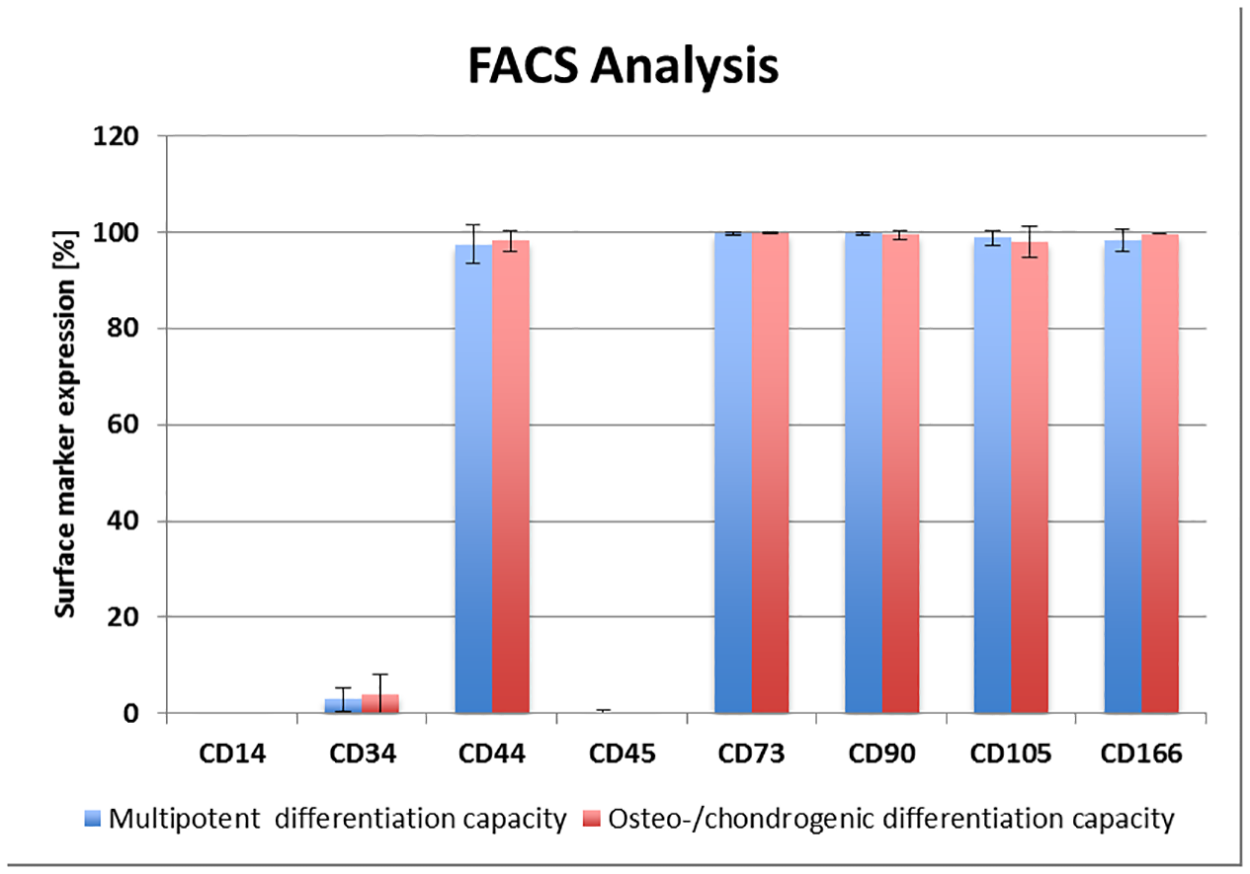
FACS-analysis. Mean values of 3 clonal cultures with osteo-chondrogenic and 3 multipotent osteo-, chondro- and adipogenic differentiation potential were given. Absence of hematopoietic cell surface marker CD14, CD34, and CD45 and nearly 100% marker presentation of CD44, CD73, CD90, CD105, and CD166 in clonal cultures with a multipotent and with an osteo-/chondrogenic differentiation capacity were presented.

The FACS analysis revealed a uniform expression of the chosen surface marker. The FITC labelled CD105, CD90 and CD44 antibodies and the PE labelled CD73 and CD166 antibodies revealed the presence (nearly 100%) of the corresponding surface marker in clonal cultures able to differentiate to the osteo-chondrogenic lineage and in multipotent clonal cultures able to differentiate to the osteo-, chondro- and adipogenic lineage. Hematopoietic marker CD45 (FITC labelled antibodies), CD14, and CD34 (PE labelled antibodies) were not detected in any culture. Due to a limited number of cells in clonal cultures FACS analysis could only be performed for 3 undifferentiated clonal cultures of osteo-chondrogenic lineage and multipotent osteo-, chondro- and adipogenic lineage potential and mean values of each were given in Fig 4.

### Genome-wide gene expression profiling

Genome-wide gene expression profiling using Affymetrix HG-U133 plus 2.0 array of 21 undifferentiated clonal cultures with different differentiation capacity (10 tripotent, 8 osteo-/chondrogenically inducible, 2 osteo-/adipogenically inducible, and osteogenically inducible) demonstrated nearly no significantly different expression of genes considering undifferentiated clonal cultures of cells with a multilineage differentiation potential and undifferentiated clonal cultures that only differentiate towards the osteogenic and chondrogenic lineage except for 7 genes (Table 6). Five of them were higher expressed in tripotent cultures and 2 of them in cultures with the potential to differentiate only into the osteogenic and chondrogenic lineage. Gene expression of *CUG Triplet Repeat*, *RNA Binding Protein 2* (*CUGBP2*) and *Kinesin Family Member 20A* (*KIF20A*) showed the highest and the lowest mean fold change (FC_m_). Furthermore, 52 genes showed a difference when comparing undifferentiated clonal culture with a multilineage potential and undifferentiated clonal cultures with the ability to develop towards the osteogenic and adipogenic lineage (Table 7). Here, 25 genes were higher express in cultures lacking the chondogenic differentiation potential and 27 genes in cultures able to differentiate towards the chondrogenic lineage. For changes in clonal cultures with different chondrogenic potential *Neural Precursor Cell expressed*, *developmentally down-regulated 9* (*NEDD9*) (+) and *Thymic Stromal Lymphopoietin* (*TSLP*) (-) showed the highest and the lowest FC_m_.

**Table 6:** Differentially expressed genes in undifferentiated clonal cultures with or without adipogenic differentiation potential.

Affymetrix ID	Symbol	Mean—Signal O/C	Mean—Signal O/C/A	FC_m_ GCOS	FC_m_ RMA	Name
**202157_s_at**	*CUGBP2*	733,48	407,31	2,91	3,05	*CUG Triplet Repeat*, *RNA Binding Protein 2*
**218574_s_at**	*LMCD1*	685,58	345,19	2,27	2,16	*LIM and Cysteine-rich Domains 1*
**232914_s_at**	*SYTL2*	251,69	377,04	-2,03	-2,19	*Synaptotagmin-like 2*
**202503_s_at**	*KIAA0101*	1 413,97	3 007,28	-2,83	-3,12	*KIAA0101*
**214710_s_at**	*CCNB1*	427,12	1 598,34	-4,32	-5,13	*Cyclin B1*
**209773_s_at**	*RRM2*	431,52	1 309,91	-4,79	-4,76	*Ribonucleotide Reductase M2 Polypeptide*
**218755_at**	*KIF20A*	131,12	523,70	-7,21	-6,15	*Kinesin Family Member 20A*

Comparison of the average signals values (Mean—Signal) of the bipotent (O/C) and the tripotent (O/C/A) samples, and their average *Fold Changes* (Mean—FC) according to GCOS and RMA analysis. O: Osteogenesis, C: Chondrogenesis, A: Adipogenesis.

**Table 7:** Differentially expressed genes in undifferentiated clonal cultures with or without chondrogenic differentiation potential.

Affymetrix ID	Symbol	Mean—Signal O/A und O	Mean—Signal O/C/A und O/C	FC_m_ GCOS	FC_m_ RMA	Name
**202149_at**	*NEDD9*	265.8	52.4	9.85	7.46	*Neural Precursor Cell expressed*, *developmentally down-regulated 9*
**228885_at**	*MAMDC2*	259.2	44.2	6.50	6.50	*MAM Domain containing 2*
**207302_at**	*SGCG*	241.8	58.5	6.50	3.48	*Sarcoglycan*, *gamma*
**219179_at**	*DACT1*	712.1	135.8	5.28	5.66	*Dapper*, *Antagonist of Beta-Catenin*, *Homolog 1*
**207030_s_at**	*CSRP2*	591.5	198.6	3.73	3.73	*Cysteine and Glycine-rich Protein 2*
**230250_at**	*PTPRB*	126.4	45.0	3.73	3.25	*Protein tyrosine phosphatase*, *receptor type*, *B*
**204470_at**	*CXCL1*	533.3	200.9	3.25	3.03	*Chemokine (C-X-C motif) Ligand 1*
**205207_at**	*IL6*	740.8	294.3	3.25	3.03	*Interleukin 6*
**210511_s_at**	*INHBA*	343.6	127.4	3.25	2.83	*Inhibin*, *beta A*
**203440_at**	*CDH2*	1 647.8	592.7	3.03	3.03	*Cadherin 2*,*Type 1*, *N-Cadherin*
**209101_at**	*CTGF*	8 166.7	3 518.4	3.03	3.03	*Connective Tissue Growth Factor*
**218469_at**	*GREM1*	4 784.2	2 113.1	3.03	3.03	*Gremlin 1*
**205518_s_at**	*CMAH*	231.6	116.5	2.83	3.25	*Cytidine Monophosphate-N-acetylneuraminic Acid Hydroxylase*
**210002_at**	*GATA6*	1 186.7	513.2	2.83	3.03	*GATA Binding Protein 6*
**205442_at**	*MFAP3L*	111.2	42.6	2.83	2.14	*Microfibrillar-associated Protein 3-like*
**208447_s_at**	*PRPS1*	1 648.0	682.1	2.46	2.46	*Phosphoribosyl Pyrophosphate Synthetase 1*
**228367_at**	*ALPK2*	1 442.6	720.4	2.30	2.30	*Alpha-Kinase 2*
**201631_s_at**	*IER3*	1 641.3	863.3	2.30	2.14	*Immediate Early Response 3*
**212530_at**	*NEK7*	4 562.5	2 134.5	2.30	2.30	*NIMA -related Kinase 7*
**215253_s_at**	*RCAN1*	796.9	386.7	2.30	2.14	*regulator of calcineurin 1*
**205807_s_at**	*TUFT1*	475.2	223.7	2.30	2.30	*Tuftelin 1*
**206085_s_at**	*CTH*	272.1	145.6	2.14	2.14	*Cystathionase*
**204421_s_at**	*FGF2*	1 015.8	519.3	2.14	2.14	*Fibroblast Growth Factor 2*
**202619_s_at**	*PLOD2*	2 658.6	1 398.6	2.14	2.14	*Procollagen-Lysine*, *2-Oxoglutarate 5-Dioxygenase 2*
**201107_s_at**	*THBS1*	1 272.2	581.0	2.14	2.00	*Thrombospondin 1*
**203186_s_at**	*S100A4*	2 523.2	6 104.7	-2.00	-2.00	*S100 Calcium Binding protein A4*
**209335_at**	*DCN*	563.8	1 526.9	-2.14	-2.00	*Decorin*
**216594_x_at**	*AKR1C1*	1 657.2	4 124.3	-2.30	-2.14	*Aldo-keto Reductase Family 1*, *Member C1*
**209160_at**	*AKR1C3*	1 649.1	3 848.2	-2.30	-2.30	*Aldo-keto Reductase Family 1*, *Member C3*
**212067_s_at**	*C1R*	1 051.0	3 300.1	-2.30	-2.14	*Complement Component 1*, *r Subcomponent*
**209699_x_at**	*AKR1C2*	1 451.4	3 870.0	-2.46	-2.30	*Aldo-keto Reductase Family 1*, *Member C2*
**204731_at**	*TGFBR3*	441.4	1 138.6	-2.46	-2.46	*Transforming Growth Factor*, *beta* *Receptor III*
**201367_s_at**	*ZFP36L2*	127.8	597.9	-2.46	-2.30	*Zinc Finger Protein 36*, *C3H Type-like 2*
**213004_at**	*ANGPTL2*	231.7	677.4	-2.64	-2.64	*Angiopoietin-like 2*
**206481_s_at**	*LDB2*	158.2	492.7	-2.64	-2.64	*LIM Domain Binding 2*
**205907_s_at**	*OMD*	43.7	140.9	-2.64	-3.25	*Osteomodulin*
**209598_at**	*PNMA2*	408.8	1 189.7	-2.64	-3.03	*Paraneoplastic Antigen MA2*
**206631_at**	*PTGER2*	145.2	456.6	-2.83	-2.83	*Prostaglandin E Receptor 2*
**227752_at**	*SPTLC3*	57.9	185.3	-2.83	-2.64	*Serine Palmitoyltransferase*, *long Chain Base Subunit 3*
**207177_at**	*PTGFR*	90.1	422.1	-3.03	-2.83	*Prostaglandin F Receptor*
**209596_at**	*MXRA5*	106.9	439.1	-3.48	-3.25	*Matrix-Remodelling associated 5*
**200986_at**	*SERPING1*	613.4	2 053.5	-3.48	-3.25	*Serpin Peptidase Inhibitor*, *Clade G*, *Member 1*
**209960_at**	*HGF*	63.5	289.5	-4.00	-4.59	*Hepatocyte Growth Factor*
**209348_s_at**	*MAF*	25.6	154.7	-4.29	-3.48	*v-maf musculoaponeurotic Fibrosarcoma Oncogene Homolog*
**217525_at**	*OLFML1*	233.7	946.4	-4.29	-0.22	*Olfactomedin-like 1*
**203666_at**	*CXCL12*	180.2	878.8	-4.59	-3.48	*Chemokine (C-X-C motif) Ligand 12*
**214022_s_at**	*IFITM1*	457.6	2 083.9	-4.59	-4.29	*Interferon induced Transmembrane Protein 1 (9–27)*
**207761_s_at**	*METTL7A*	182.4	912.2	-4.59	-4.29	*Methyltransferase like 7A*
**213493_at**	*SNED1*	68.4	457.9	-4.92	-3.48	*Sushi*, *Nidogen and EGF-like Domains 1*
**229839_at**	*SCARA5*	82.6	584.6	-8.57	-5.28	*Scavenger Receptor Class A*, *Member 5*
**201427_s_at**	*SEPP1*	68.9	832.6	-9.19	-9.19	*Selenoprotein P*, *Plasma*, *1*
**235737_at**	*TSLP*	36.2	380.7	-9.85	-8.57	*Thymic Stromal Lymphopoietin*

Comparison of the average signal values (Mean—Signal) of cultures with (O/C/A and O/C) and without (O/A and O) adipogenic differentiation potential and their average *Fold Changes* (Mean—FC) according to GCOS and RMA analysis. O: Osteogenesis, C: Chondrogenesis, A: Adipogenesis.

## Discussion

The main goal of tissue engineering is to regenerate tissue or organ defects by means of cell suspensions, cell-based transplants or by chemotactically attracting cells into the defect. Regulatory authorities, such as the Food and Drug Administration and the European Medicines Agency, have established strict regulations for the approval and use of such therapies in relation to standards in the area of warranty, product quality and product safety. For the use of cell-based products in clinical use, it is therefore essential to characterize the used cells extensively. Among other things, questions such as the potential of the cells as well as the delimitation to other tissues play an important role.

Progenitor cells from the periosteum are promising candidates for the application in the regeneration of bones and cartilage. They are able to differentiate *in vitro* into bones, cartilage and fat and exhibit similar properties, such as mesenchymal stem cells or multipotent mesenchymal stromal cells of bone marrow [9]. Furthermore, the rate of *in vivo* bone formation of canine periosteal cells was compared to alveolar osteoblasts and bone marrow-derived MSC much higher underlining the potential for bone formation [13]. Periosteum-derived progenitor cells are already in clinical use for the regeneration of upper jaw bones [2, 4, 14]. Nevertheless, the question remains whether all cells derived from the periosteum are able to differentiate in all lineages or if there are subpopulations. In the latter case, a cell sorting could be of interest to ensure cell purity and efficacy of the cells for clinical applications. In order to analyze the differentiation potential of these cells and to investigate different subclonal populations, clonal cultures of periosteal cells were developed and differentiated into bone, cartilage and fat. In addition, clonal cultures were analyzed by flow cytometry and genome-wide microarrays of undifferentiated cells to determine their gene expression profiles. Based on microarray results, important similarities and differences in different subtypes of clonal cultures were demonstrated.

To determine the common or different potential of periosteal cells, clonal cultures were applied from single cells isolated from the periosteum with a modified procedure already described [10, 15]. Other possible techniques are to generate clonal cultures by dilution of isolated cells to such an extent that theoretically only one cell is present in a defined volume which is seeded into a well 96-well plate [8, 16]. The disadvantage with this technique, however, is that the singulation is only a theoretical assumption and more than one cell could easily be located in one well. Also CellTracker labeling of freshly isolated cells as a tool of for identification of single cells in well plates [17] was avoided to prevent cell stress and loss of the very limited number of periosteal cells after cell isolation.

As also described for bone marrow MSC [10, 16, 17], the growth and differentiation potential of clonal cultures was heterogeneous. We found both, clonal cultures that did not grew or did very slowly, and thus had no or only very limited expansion potential, as well as fast-growing clonal cultures, which led to over 1 x 10^6^ cells after 3 weeks. In a mixed population, the fast-growing cells would rapidly displace the slowers. In previous publications of clonal periosteal cultures only those with a multiple differentiation potential have been described. Thus, DeBari generated seven clonal cultures from four donors, all of which had the potential to differentiate into the adipogenic, chondrogenic and osteogenic direction. Even an *in vivo* formation of skeletal muscle could be induced [8]. The results obtained in the work presented here, showed for the first time that there are also periosteal cells with a more limited potential. A similar spectrum of multi-, bi- or monopotent cells was found in Muraglia et al., where 185 clonal MSC cultures were examined [16]. There, the proportion of multipotent cells differentiating in all tested lineages was 24.3% and therefore lower than in the clonal cultures derived from the periosteum of the mastoid (49%). Cells with the potential to differentiate only in the osteo- and adipogenic direction were not found in MSC cultures. In contrast to the MSC, there were no clonal periosteal cultures that could not be induced at all. In contrast to Muraglia et al., a different study demonstrated a 50% rate of tri-lineage potent clonal MSC cell cultures [17] similar to the clonal periosteal cultures found here. But in contrast to our results and Muraglia et al. clonal cultures with a single adipogenic and chondrogenic were detected. When comparing the ostegenic differentiation capacity of periosteal clonal cultures to those of MSC, it is shown that there are cells in the heterogenous clonal MSC cultures that display no capability to differentiate to the osteogenic lineage [16, 17]. On the other hand, 100% of the periosteal cells showed an osteogenic potential. In regard towards a clinical application in bone regeneration periosteal cells seem to be more promising than MSC from bone marrow. No cell sorting prior application is needed when all cells are able to differentiate to the desired cell lineage. Overall, 94.1% of clonal cultures with a chondrogenic potential were found. This is also similar (94%) to published results for bone marrow-derived clonal MSC cultures [16]. For adipogenic differentiation a higher amount of clonal cultures of periosteal progenitor cells than known from the literature for bone marrow-derived MSC was able to differentiate (52.9% compared to 28.6%) [16].

In addition to the analysis of the differentiation capacity of our clonal cultures we tried to compare them with their corresponding growth characteristics. We did not find any correlations. Clonal cultures from all growth classes (fast, mean or slow growth rates, low, mean or high poulation doubling times) were able to differentiate and others were not able to do so.

To distinguish between clonal cultures with different differentiation potential their undifferentiated gene expression profiles were correlated. Genome—wide microarrays of 21 undifferentiated clonal cultures were compared in order to find potentially predictive gene expression patterns. However, when comparing tripotent cells with chondrogenic and osteogenic inducible cells, only 7 genes were differentially expressed with *CUGBP2* and *KIF20A* containing the widest range of FC_m_ (+/-) between the two groups of clonal cultures. A distinction between these two groups on the basis of these 7 genes was not possible. The differences comparing cells with or without chondrogenic differentiation potential with 52 differentially expressed genes were low. *NEDD9* showed the highest FC_m_ for gene expression of undifferentiated clonal cultures that could not be differentiated towards the chondrogenic lineage compared to tripotent clonal cultures. *NEDD9* is described to be a scaffolding protein in the integrin signaling pathway that is involved in cell adhesion dynamics [18]. The gene expression for *TSLP* displayed the highest FC_m_ in undifferentiated tripotent clonal cultures compared cultures that did not comprise chondrogenic differentiation. For keloid firboblasts *TSLP* is described as a potent inducer of collagen and TGF-β production [19]. Among the other 52 genes with different gene expression is *Transforming Growth Factor beta Type III Receptor* (*TGFBR3*). It encodes the receptor with the same name. The receptor plays an important role for TGF-β and BMP signaling [20]. Furthermore, the gene expression of *Decorin* (*DCN*) encoding the cartilage protein decorin [21] was higher in chondrogenically inducible cultures. Among the genes higher expressed in clonal cultures not able to form cartilage was *Gremlin 1* (*GREM1*). The corresponding protein is a BMP antagonist which was found in higher concentrations in early osteoarthritic cartilage [22]. The reduced expression of *DCN*, *TGFBR3*, *TSLP* and the increased expression of *GREM1* in clonal cultures not able to induce chondrogenesis might be an interesting chondrogenic indicator.

A further confirmation of similarity between the two groups of tripotent cells and clonal cultures not able to induce adipogenic differentiation was provided by FACS analysis. Although no specific marker is known for periosteal cells, a set of surface marker that is also used to characterize MSC (CD166, CD105, CD90, CD73, CD44 positive and CD45, CD34, CD14 negative) was chosen. FACS analysis did not show any differences between the two groups and demonstrated the same results as a whole population of periosteal progenitor cells without clonal cultures [9, 23, 24].

The evaluation of clonal cultures of periosteum-derived cells revealed that there are subpopulations with different differentiation capacities. But all generated clonal cultures were able to differentiate into the osteogenic lineage. Therefore, for a clinical application of periosteal cells in bone regeneration all prerequisites in case of cell potency are given. For the application of periosteal progenitor cells in cartilage regeneration a small subpopulation is not able be induced to the chondrogenic lineage. Here, gene expressions of *DCN*, *GREM1*, *NEDD9*, *TGFBR3*, and *TSLP* seem to be interesting candidates to distinguish between cell populations with different chondrogenic differentiation capacities. The display of clonal cultures with or without adipogenic differentiation capacity has no impact on potential clinical applications.

## Supporting information

S1 Table Growth of clonal cultures. Growth of clonal cultures with max. passage number, max. theoretical cell number, population doublings, average growth rate, population doubling time, and growth class.
(DOCX)

S2 Table Overview of the histologic and immunohistochemic stainings. Osteogenic (alkaline phosphatase and von Kossa staining), chondrogenic (Alcian blue 8GX staining and immunohistochemical collagen type II staining), and adipogenic differentiation (oil red O staining) of clonal cultures. “-”no staining, “(+)”weak staining, “+”positive staining, “++”strong staining, “C”non-induced control und “I”induced.
(DOCX)
