# Supplementary material for: Characterization of single cell derived cultures of periosteal progenitor cells to ensure the cell quality for clinical application
Source: PLoS One. 2017 May 31;12(5):e0178560. doi: 10.1371/journal.pone.0178560 (PMC5451110; doi:10.1371/journal.pone.0178560)
Supplement: S1 Table — Growth of clonal cultures with max. passage number, max. theoretical cell number, population doublings, average growth rate, population doubling time, and growth class. (DOCX) [file pone.0178560.s001.docx]

**S1 Table. Growth of clonal cultures.**

| **Donor** | **Clonal culture** | **Passage number P_max_** | **Max. theor. cell number** | **Population doublings** | **Average growth rate**  **µ [1/d]** | **Population doubling time [d]** | **Growth class** |
| --- | --- | --- | --- | --- | --- | --- | --- |
| **Donor 1** | **Cl1** | 6 | 3.78 x 10^6^ | 21.8 | 0.211 | 3,3 | 5 |
|  | **Cl2** | 6 | 2.60 x 10^7^ | 24.6 | 0.217 | 3,2 | 5 |
|  | **Cl3** | 5 | 2.92 x 10^6^ | 21.5 | 0.105 | 6,6 | 6 |
|  | **Cl4** | 4 | 2.40 x 10^5^ | 17.9 | 0.175 | 4,0 | 8 |
|  | **Cl5** | 8 | 5.14 x 10^7^ | 25.6 | 0.157 | 4,4 | 5 |
|  | **Cl6** | 9 | 4.11 x 10^8^ | 28.6 | 0.177 | 3,9 | 2 |
|  | **Cl7** | 9 | 1.75 x 10^9^ | 30.7 | 0.162 | 4,3 | 2 |
|  | **Cl8** | 6 | 1.79 x 10^7^ | 24.1 | 0.200 | 3,5 | 5 |
| **Donor 2** | **Cl1** | 9 | 3.08 x 10^9^ | 31.5 | 0.246 | 2,8 | 1 |
|  | **Cl2** | 8 | 1.46 x 10^8^ | 27.1 | 0.199 | 3,5 | 5 |
|  | **Cl3** | 6 | 1.76 x 10^7^ | 24.1 | 0.220 | 3,2 | 5 |
|  | **Cl4** | 7 | 2.08 x 10^8^ | 27.6 | 0.197 | 3,5 | 5 |
|  | **Cl5** | 5 | 2.13 x 10^6^ | 21.0 | 0.201 | 3,5 | 5 |
|  | **Cl6** | 6 | 5.05 x 10^7^ | 25.6 | 0.207 | 3,3 | 5 |
|  | **Cl7** | 5 | 3.86 x 10^6^ | 21.9 | 0.089 | 7,8 | 6 |
|  | **Cl8** | 7 | 5.05 x 10^7^ | 25.6 | 0.150 | 4,6 | 5 |
|  | **Cl9** | 10 | 7.21 x 10^9^ | 32.7 | 0.209 | 3,3 | 2 |
|  | **Cl10** | 9 | 2.20 x 10^9^ | 31.0 | 0.197 | 3,5 | 2 |
|  | **Cl11** | 5 | 4.38 x 10^6^ | 22.1 | 0.146 | 4,8 | 6 |
|  | **Cl12** | 5 | 8.25 x 10^5^ | 19.7 | 0.056 | 12,4 | 6 |
|  | **Cl13** | 6 | 1.85 x 10^7^ | 24.1 | 0.179 | 3,9 | 5 |
|  | **Cl14** | 6 | 3.90 x 10^6^ | 21.9 | 0.130 | 5,3 | 6 |
|  | **Cl15** | 9 | 1.00 x 10^9^ | 29.9 | 0.231 | 3,0 | 1 |
|  | **Cl16** | 8 | 7.27 x 10^7^ | 26.1 | 0.169 | 4,1 | 5 |
|  | **Cl17** | 6 | 1.33 x 10^7^ | 23.7 | 0.116 | 6,0 | 6 |
|  | **Cl18** | 5 | 1.08 x 10^7^ | 23.4 | 0.117 | 5,9 | 6 |
|  | **Cl19** | 8 | 6.37 x 10^7^ | 25.9 | 0.144 | 4,8 | 6 |
|  | **Cl20** | 6 | 9.42 x 10^7^ | 26.5 | 0.213 | 3,2 | 5 |
|  | **Cl21** | 7 | 1.82 x 10^8^ | 27.4 | 0.328 | 2,1 | 4 |
| **Donor 3** | **Cl1** | 7 | 3.44 x 10^7^ | 25.0 | 0.231 | 3,0 | 4 |
|  | **Cl2** | 7 | 2.96 x 10^8^ | 28.1 | 0.230 | 3,0 | 4 |
|  | **Cl3** | 6 | 7.72 x 10^6^ | 22.9 | 0.281 | 2,5 | 4 |
|  | **Cl4** | 4 | 1.10 x 10^5^ | 16.7 | 0.075 | 9,2 | 9 |
|  | **Cl5** | 8 | 3.19 x 10^9^ | 31.6 | 0.320 | 2,2 | 4 |
|  | **Cl6** | 5 | 9.25 x 10^5^ | 19.8 | 0.038 | 18,0 | 6 |
|  | **Cl7** | 7 | 9.56 x 10^7^ | 26.5 | 0.194 | 3,6 | 5 |
|  | **Cl8** | 7 | 2.45 x 10^7^ | 24.5 | 0.148 | 4,7 | 6 |
|  | **Cl9** | 8 | 2.59 x 10^8^ | 27.9 | 0.234 | 3,0 | 4 |
|  | **Cl10** | 7 | 2.65 x 10^8^ | 28.0 | 0.349 | 2,0 | 4 |
|  | **Cl11** | 6 | 1.21 x 10^8^ | 26.8 | 0.269 | 2,6 | 4 |
|  | **Cl12** | 6 | 3.33 x 10^7^ | 25.0 | 0.239 | 2,9 | 4 |
|  | **Cl13** | 9 | 1.35 x 10^9^ | 30.3 | 0.263 | 2,6 | 1 |
|  | **Cl14** | 4 | 1.48 x 10^6^ | 20.5 | 0.093 | 7,5 | 9 |
|  | **Cl15** | 6 | 9.42 x 10^6^ | 23.2 | 0.118 | 5,9 | 6 |
|  | **Cl16** | 9 | 3.59 x 10^8^ | 28.4 | 0.188 | 3,7 | 2 |
|  | **Cl17** | 4 | 5.10 x 10^5^ | 19.0 | 0.053 | 13,0 | 9 |
|  | **Cl18** | 4 | 5.80 x 10^5^ | 19.1 | 0.080 | 8,6 | 9 |
|  | **Cl19** | 8 | 1.57 x 10^8^ | 27.2 | 0.223 | 3,1 | 4 |
|  | **Cl20** | 4 | 9.00 x 10^6^ | 23.1 | 0.349 | 2,0 | 7 |
|  | **Cl21** | 9 | 8.81 x 10^8^ | 29.7 | 0.243 | 2,8 | 1 |
|  | **Cl22** | 8 | 2.54 x 10^8^ | 27.9 | 0.290 | 2,4 | 4 |
|  | **Cl23** | 5 | 9.86 x 10^6^ | 23.2 | 0.236 | 2,9 | 4 |
|  | **Cl24** | 4 | 1.03 x 10^6^ | 20.0 | 0.289 | 2,4 | 7 |
|  | **Cl25** | 7 | 5.67 x 10^7^ | 25.8 | 0.232 | 3,0 | 4 |
| **Donor 4** | **Cl1** | 7 | 5.26 x 10^7^ | 25.6 | 0.223 | 3,1 | 4 |
|  | **Cl2** | 8 | 7.77 x 10^7^ | 26.2 | 0.282 | 2,5 | 4 |
|  | **Cl3** | 7 | 4.66 x 10^7^ | 25.5 | 0.215 | 3,2 | 5 |
|  | **Cl4** | 6 | 8.97 x 10^6^ | 23.1 | 0.174 | 4,0 | 5 |
|  | **Cl5** | 5 | 4.90 x 10^6^ | 22.2 | 0.196 | 3,5 | 5 |
|  | **Cl6** | 5 | 8.64 x 10^5^ | 19.7 | 0.120 | 5,8 | 6 |
|  | **Cl7** | 5 | 1.34 x 10^6^ | 20.4 | 0.100 | 7,0 | 6 |
|  | **Cl8** | 6 | 3.23 x 10^6^ | 21.6 | 0.099 | 7,0 | 6 |
|  | **Cl9** | 6 | 4.00 x 10^7^ | 25.3 | 0.264 | 2,6 | 4 |
|  | **Cl10** | 5 | 4.99 x 10^5^ | 18.9 | 0.096 | 7,2 | 6 |
|  | **Cl11** | 13 | 7.34 x 10^10^ | 36.1 | 0.274 | 2,5 | 1 |
|  | **Cl12** | 6 | 1.35 x 10^7^ | 23.7 | 0.201 | 3,4 | 5 |
|  | **Cl13** | 9 | 2.93 x 10^8^ | 28.1 | 0.157 | 4,4 | 2 |
|  | **Cl14** | 5 | 1.92 x 10^6^ | 20.9 | 0.164 | 4,2 | 5 |
|  | **Cl15** | 6 | 8.42 x 10^6^ | 23.0 | 0.215 | 3,2 | 5 |
|  | **Cl16** | 5 | 1.37 x 10^7^ | 23.7 | 0.233 | 3,0 | 4 |
|  | **Cl17** | 4 | 4.40 x 10^5^ | 18.7 | 0.085 | 8,2 | 9 |
|  | **Cl18** | 6 | 4.30 x 10^7^ | 25.4 | 0.274 | 2,5 | 4 |
|  | **Cl19** | 5 | 6.12 x 10^5^ | 19.2 | 0.075 | 9,3 | 6 |
|  | **Cl20** | 3 | 1.50 x 10^5^ | 17.2 | 0.018 | 38,8 | 9 |
|  | **Cl21** | 5 | 4.10 x 10^5^ | 18.6 | 0.144 | 4,8 | 6 |
|  | **Cl22** | 5 | 7.38 x 10^5^ | 19.5 | 0.104 | 6,7 | 6 |
|  | **Cl23** | 4 | 2.30 x 10^5^ | 18.2 | 0.134 | 5,2 | 9 |
|  | **Cl24** | 4 | 1.80 x 10^5^ | 17.5 | 0.107 | 6,5 | 9 |
|  | **Cl25** | 5 | 4.82 x 10^5^ | 18.9 | 0.062 | 11,1 | 6 |
|  | **Cl26** | 4 | 3.20 x 10^5^ | 18.3 | 0.180 | 3,8 | 8 |
|  | **Cl27** | 4 | 2.30 x 10^5^ | 17.8 | 0.109 | 6,3 | 9 |
|  | **Cl28** | 4 | 3.60 x 10^5^ | 18.5 | 0.095 | 7,3 | 9 |
|  | **Cl29** | 5 | 3.47 x 10^5^ | 18.4 | 0.090 | 7,7 | 6 |
