# Supplementary material for: Characterization of single cell derived cultures of periosteal progenitor cells to ensure the cell quality for clinical application
Source: PLoS One. 2017 May 31;12(5):e0178560. doi: 10.1371/journal.pone.0178560 (PMC5451110; doi:10.1371/journal.pone.0178560)
Supplement: S2 Table — Osteogenic (alkaline phosphatase and von Kossa staining), chondrogenic (Alcian blue 8GX staining and immunohistochemical collagen type II staining), and adipogenic differentiation (oil red O staining) of clonal cultures. “-”no staining, “(+)”weak staining, “+”positive staining, “++”strong staining, “C”non-induced control und “I”induced. (DOCX) [file pone.0178560.s002.docx]

**S2 Table. Overview of the histologic and immunohistochemic stainings.**

| **Donor** | **Clonal culture** | **Growth class** | **Chondrogenic Differentiation** | | | | **Osteogenic Differentiation** | | | | **Adipogenic Differentiation** | |
| --- | --- | --- | --- | --- | --- | --- | --- | --- | --- | --- | --- | --- |
|  |  |  | **Alkaline phosphatase staining** | | **Von Kossa staining** | | **Alcian blue 8GX staining** | | **Collagen type II staining** | | **Adipo Histo Oil Red O** | |
|  |  |  | **Day 28C** | **Day 28I** | **Day 28C** | **Day 28I** | **Day 28C** | **Day 28I** | **Day 28C** | **Day 28I** | **Day 15C** | **Day 15I** |
| **Donor 1** | **Cl2** | 5 | (+) | + | - | ++ | + | ++ | - | ++ | (+) | ++ |
|  | **Cl6** | 2 | (+) | + | - | ++ | + | ++ | - | ++ | (+) | (+) |
|  | **Cl7** | 2 | + | ++ | - | + | + | ++ | - | + | (+) | (+) |
| **Donor 2** | **Cl1** | 1 | detached | + | - | + | + | ++ | (+) | ++ | (+) | + |
|  | **Cl2** | 5 | (+) | + | - | + | + | ++ | - | ++ | (+) | - |
|  | **Cl3** | 5 | (+) | + | - | ++ | (+) | + | - | (+) | (+) | ++ |
|  | **Cl4** | 5 | (+) | + | - | + | (+) | ++ | - | (+) | (+) | ++ |
|  | **Cl5** | 5 | - | + | - | + | (+) | ++ | - | ++ | (+) | ++ |
|  | **Cl6** | 5 | - | + | - | ++ | (+) | ++ | - | ++ | (+) | + |
|  | **Cl7** | 6 | + | + | - | ++ | + | - | - | - | (+) | + |
|  | **Cl8** | 5 | (+) | + | - | + | + | ++ | (+) | + | - | - |
|  | **Cl9** | 2 | (+) | + | - | ++ | (+) | ++ | (+) | ++ | (+) | + |
|  | **Cl10** | 2 | - | + | - | + | (+) | ++ | - | + | (+) | + |
|  | **Cl11** | 6 | + | + | - | ++ | (+) | ++ | - | ++ | - | - |
|  | **Cl13** | 5 | (+) | + | - | + | - | + | - | ++ | - | - |
|  | **Cl15** | 1 | (+) | + | - | ++ | + | ++ | - | ++ | - | - |
|  | **Cl16** | 5 | (+) | + | - | ++ | (+) | + | (+) | + | (+) | (+) |
|  | **Cl17** | 6 | (+) | + | - | ++ | (+) | ++ | (+) | ++ | (+) | (+) |
|  | **Cl18** | 6 | (+) | + | - | ++ | (+) | ++ | (+) | ++ | + | ++ |
|  | **Cl19** | 6 | - | + | - | ++ | (+) | ++ | - | ++ | (+) | + |
|  | **Cl20** | 5 | - | + | - | ++ | (+) | ++ | - | ++ | (+) | - |
|  | **Cl21** | 4 | + | + | - | + | (+) | ++ | - | (+) | (+) | ++ |
| **Donor 3** | **Cl1** | 4 | (+) | + | - | + | (+) | ++ | - | + | - | - |
|  | **Cl2** | 4 | - | + | - | + | + | ++ | - | + | - | + |
|  | **Cl3** | 4 | (+) | + | - | ++ | (+) | ++ | - | + | (+) | - |
|  | **Cl5** | 4 | - | + | - | + | (+) | ++ | - | + | - | + |
|  | **Cl7** | 5 | - | + | - | ++ | (+) | ++ | - | + | - | + |
|  | **Cl8** | 6 | (+) | + | - | ++ | + | ++ | - | + | - | - |
|  | **Cl9** | 4 | - | + | - | ++ | + | ++ | - | ++ | - | ++ |
|  | **Cl10** | 4 | (+) | + | - | ++ | (+) | ++ | - | + | (+) | + |
|  | **Cl11** | 4 | (+) | + | - | + | + | ++ | - | + | - | - |
|  | **Cl12** | 4 | (+) | + | - | ++ | ++ | ++ | - | + | - | - |
|  | **Cl13** | 1 | (+) | + | - | ++ | + | ++ | - | ++ | (+) | - |
|  | **Cl15** | 6 | - | + | - | ++ | + | ++ | - | + | - | - |
|  | **Cl16** | 2 | (+) | + | - | ++ | + | ++ | - | + | - | - |
|  | **Cl19** | 4 | (+) | + | - | ++ | + | ++ | - | - | - | (+) |
|  | **Cl20** | 7 | (+) | + | - | ++ | + | ++ | - | + | - | - |
|  | **Cl21** | 1 | (+) | + | - | ++ | + | - | - | - | - | - |
|  | **Cl22** | 4 | (+) | + | - | ++ | + | ++ | - | ++ | - | + |
|  | **Cl23** | 4 | (+) | + | - | (+) | (+) | ++ | - | + | - | - |
|  | **Cl25** | 4 | - | + | - | ++ | + | ++ | - | + | - | - |
| **Donor 4** | **Cl1** | 4 | - | + | - | ++ | (+) | ++ | - | + | (+) | + |
|  | **Cl4** | 5 | (+) | + | - | + | + | ++ | - | + | (+) | + |
|  | **Cl5** | 5 | (+) | + | - | ++ | + | ++ | - | ++ | (+) | ++ |
|  | **Cl9** | 4 | (+) | + | - | + | + | ++ | - | ++ | (+) | ++ |
|  | **Cl11** | 1 | (+) | + | - | ++ | + | ++ | - | ++ | (+) | + |
|  | **Cl12** | 5 | (+) | + | - | ++ | + | ++ | - | + | (+) | (+) |
|  | **Cl13** | 2 | (+) | + | - | ++ | + | ++ | - | + | + | ++ |
|  | **Cl15** | 5 | (+) | + | - | + | + | ++ | - | + | (+) | + |
|  | **Cl16** | 4 | (+) | + | - | ++ | - | ++ | - | ++ | (+) | (+) |
|  | **Cl18** | 4 | (+) | + | - | ++ | (+) | ++ | - | ++ | (+) | + |

**Osteogenic (alkaline phosphatase and von Kossa staining), chondrogenic (Alcian blue 8GX staining and immunohistochemical collagen type II staining), and adipogenic differentiation (oil red O staining) of clonal cultures. “-“ no staining, “(+)“ weak staining, “+“ positive staining, “++“ strong staining, “C“ non-induced control und “I“ induced.**
